# Tracking of Mutational Signature of SARS-CoV-2 Omicron on Distinct Continents and Little Difference was Found

**DOI:** 10.3390/v15020321

**Published:** 2023-01-23

**Authors:** Shu-Yue Zheng, Yun-Peng Zhang, Yu-Xin Liu, Wei Zhao, Xiang-Lei Peng, Yan-Peng Zheng, Yuan-Hui Fu, Jie-Mei Yu, Jin-Sheng He

**Affiliations:** College of Life Sciences and Bioengineering, Beijing Jiaotong University, Beijing 100044, China

**Keywords:** Omicron variant, evolution, SNPs, mutation linkage, immune pressure

## Abstract

The Omicron variant is currently ravaging the world, raising serious concern globally. Monitoring genomic variations and determining their influence on biological features are critical for tracing its ongoing transmission and facilitating effective measures. Based on large-scale sequences from different continents, this study found that: (i) The genetic diversity of Omicron is much lower than that of the Delta variant. Still, eight deletions (Del 1–8) and 1 insertion, as well as 130 SNPs, were detected on the Omicron genomes, with two deletions (Del 3 and 4) and 38 SNPs commonly detected on all continents and exhibiting high-occurring frequencies. (ii) Four groups of tightly linked SNPs (linkage I–IV) were detected, among which linkage I, containing 38 SNPs, with 6 located in the RBD, increased its occurring frequency remarkably over time. (iii) The third codons of the Omicron shouldered the most mutation pressures, while the second codons presented the least flexibility. (iv) Four major mutants with amino acid substitutions in the RBD were detected, and further structural analysis suggested that the substitutions did not alter the viral receptor binding ability greatly. It was inferred that though the Omicron genome harbored great changes in antigenicity and remarkable ability to evade immunity, it was immune-pressure selected. This study tracked mutational signatures of Omicron variant and the potential biological significance of the SNPs, and the linkages await further functional verification.

## 1. Introduction

Since the beginning of COVID-19, five variants of severe acute respiratory syndrome coronavirus 2 (SARS-CoV-2) were designated as variants of concern (VOC) by the World Health Organization (WHO): Alpha, Beta, Gamma, Delta, and Omicron. The Omicron variant (B.1.1.529) was first identified in Botswana and South Africa in November 2021 and quickly became the predominant epidemic variant in the world within 2 months. Compared with other VOCs, Omicron is more rapidly spreading and more concealed, which brings great challenges to the prevention and control of COVID-19. It was highly mutated, and the spike glycoprotein (S) protein alone contains at least 31 mutations, while nsp4, nsp14, nsp3, membrane protein E, membrane protein M, and nuclear protein N also contain large numbers of mutations [1]. More notably, 15 of those mutations occurred in the RBD. The Omicron variant potentially exhibited increased transmissibility [2], severe antibody escape rate [3,4], and extended reduction in neutralization by post-vaccination sera [5,6]. The variant continues to evolve during transmission, and multiple epidemic sublineages have emerged: BA.1, BA.2, BA.3, BA.4, BA.5, and other descendants thereof.

The receptor-binding domain (RBD) and N-terminal domain (NTD) in the S of SARS-CoV-2 are respectively the main and the auxiliary interactions with the host cell; mutations in the domains can confer resistance against multiple neutralizing antibodies and change affinity between the virus and its receptor [7,8,9,10]. In addition, several mutations in non-S proteins have been reported to be associated with viral infectivity and replication [11]. Due to various preventive, protective or containment measures adopted, different vaccine types and coverage accessed, as well as inconsistent genetic backgrounds of populations, it is inferred that the transmission dynamics and evolution directions of SARS-CoV-2 in different global geographical regions and populations may have large variability [12]. In fact, previous studies revealed that SARS-CoV-2 exhibited epidemiological diversity and varied mutation frequency spatiotemporally; the site carrying the informative mutations are indicative for monitoring the viral transmission dynamics and evolutionary direction in different geographical regions and populations [13,14].

VOC Omicron has been circulating for nearly one year since the emergence, and it is unclear how it will establish its evolutionary pattern throughout the world. Multi-locus genome surveillance of the Omicron variant is very important for monitoring viral transmission globally, and a comprehensive understanding of global and regional variation dynamics of the virus is also critical to guide current and future interventions. In this study, by using large-scale SARS-CoV-2 Omicron genomes retrieved from the Global Initiative on Sharing Avian Influenza Data (GISAID) database [15], we uncovered variation characteristics and evolutionary scenarios for viral populations among different continents.

## 2. Materials and Methods

### 2.1. Genome Sequence Dataset Retrieval

SARS-CoV-2 genomic sequences in this study were all retrieved from GISAID by selecting the “VOC Omicron GRA (B.1.1.529+BA.*)” as the “Variants” search terms. The top 1 or 2 countries of Omicron sequence number in each continent before April 2022 were selected as the representative countries of each continent, i.e., Japan in Asia, South Africa in Africa, Portugal and England in Europe, the USA and Mexico in North America, Chile and Peru in South America, and Australia and New Zealand in Oceania. The sequences were downloaded, ensuring that no less than 15 sequences were collected from the same day and the same country. After preliminary analysis, it was found that the number of sequences in South America were relatively low, so Colombia and Peru were added as representative countries there.

### 2.2. Sequence Processing

The sequences were annotated with the accession number, clear sampling time, and location. Low-quality sequences with Ns or gaps were removed, and all the sequences were aligned by using MAFFT 7 (online version, http://mafft.cbrc.jp/alignment/software, accessed on 25 July 2021) and adjusted manually by MEGA 7 software. The Omicron genomic sequence from South Africa in the very early time (9 November 2021, EPI_ISL6913991) was used as reference for mutation analysis in this study.

### 2.3. Genetic Diversity Calculation

For the whole SARS-CoV-2 Omicron genomes, the single nucleotide polymorphisms (SNPs) calling and SNP-linkage analysis were developed, and the compiled R codes were deposited in Github as previously described [16]. Data was analyzed with GraphPad Prism (version 8.0.2).

### 2.4. Tertiary Structure-Based Affinity Prediction

To evaluate the effect of substitutions in the RBD, the interactions of the RBD with the human angiotensin-converting enzyme 2 (hACE2) in host cells were characterized using the HDOCK server (http://hdock.phys.hust.edu.cn/, accessed on 13 November 2021), based on a hybrid algorithm of template modeling with default parameters. The structures were further calculated and annotated using PyMOL software (version 2).

## 3. Results

### 3.1. Data Information

The Omicron variant sequences in this study were retrieved as of 31 August 2022. A total of 22,792 nearly full-length sequences (29,379 bp) were collected. After removing the low-quality sequences with more than 10 Ns, 9796 sequences were left, among which 1509, 1178, 1062, 1725, 2187, and 2135 were respectively downloaded from North America, Oceania, Africa, South America, Europe, and Asia. Due to unbalanced and poor-quality of sample sequencing in Africa, only South Africa had a relatively enough number of sequences, and it was the only country in Africa included in this study, so the number of sequences from Africa was the smallest; while due to the large-scale and high-quality of sequencing in Europe and Asia, their sequence numbers were relatively large. The period covered in this study was calculated every two months, and it was revealed that in the very early stage (November to December in 2021), Africa harbored the most sequences (Figure S1).

### 3.2. Deletions-and-Insertion Mutations Existed on the Omicron Genome

Nucleotide deletions and insertions with different lengths on the Omicron genomes were detected in this study. Eight deletions (Del) and 1 insertion (Inser) were found, with Del 1–3 located in the NSP1, and Del 4–8 and Inser 1 in the S. Of the deletions, Del 4 and 6 were commonly detected with high-occurring frequencies on the six continents (all were >60%); Del 2 was absent in North America and South America and had low frequencies in other continents; Del 1 was Del 2 without the leading and trailing amino acid, which exhibited a low frequency and only occurred in Africa; Del 3 occurred on all the continents except Oceania, and the frequencies ranged from 2.18% in Asia to 19.17% in Africa; Del 5 and 8 occurred simultaneously and only presented in Asia, while Del 7 occurred only in South America, and the frequencies of Del 5/8 and Del 7 were 12.89% and 13.79%, respectively. In addition, the insertion (Inser 1) added an amino acid between the amino acid position 206 and 207 on the S protein, and it only happened in Europe and Asia, with frequencies of 38.69% and 37.35%, respectively (Table 1).

**Figure 1:**
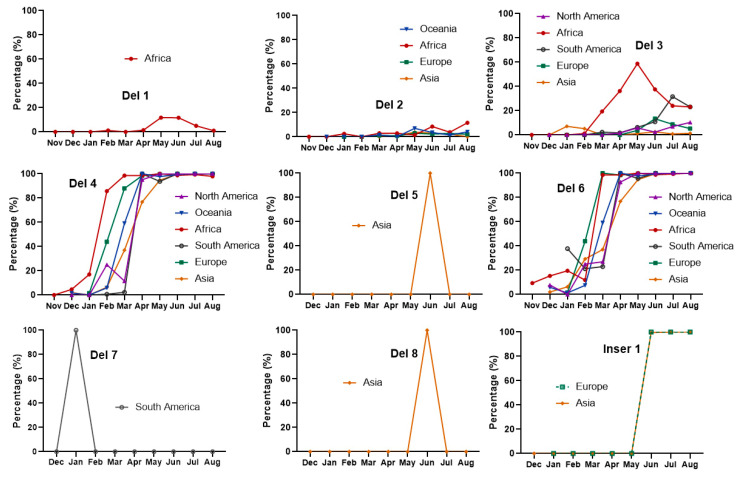
Temporal distribution of the deletions and insertions. Del 4 and 6 were commonly detected, and their occurring frequencies increased significantly. Other deletions were either of low frequencies or only transiently exist in a specific region. Inser 1 presented in Europe and Asia with 100% frequency from May 2022.

Temporal distribution of the deletions and insertions on the six continents were analyzed. To avoid bias caused by small numbers, early months with fewer than 30 sequences were excluded in this study, including all the regions except Africa in November 2021 and Asia and Europe in December 2021. Results showed that the distributions of the deletions/insertions exhibited commonality and characteristics across continents. Specifically, both Del 4 and Del 6 had low frequencies in the early stage but increased significantly over time and rapidly reached 100%. Del 3 presented a higher frequency in Africa than in other continents; the frequency increased markedly from February and reached a peak in May (with a frequency about 60%), then declined to the same level as South America. Both Del 1 and 2 exhibited low occurring frequencies in each month, and Del 1 appeared only in May to July in Africa. Del 5/8 and 7 only presented in June in Asia and in January in South America, respectively, and occurred with 100% frequency. Inser 1 started to appear in Europe and Asia in June with occurring frequency of 100% (Figure 1).

### 3.3. SNP Calling

**Figure 2:**
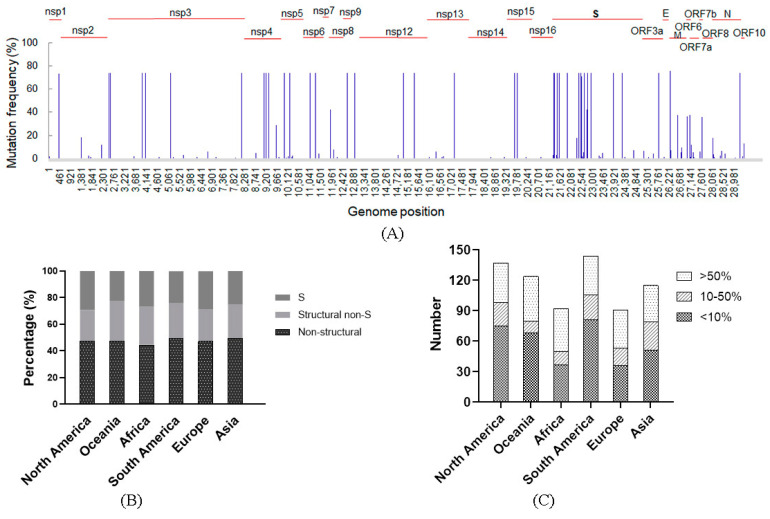
SNPs’ distributions. (**A**) Total SNPs of the sequences collected in this study. A total of 130 SNPs was found. (**B**) Gene distribution. Structural genes presented more SNPs than the non-structural genes. S gene had the most SNPs. (**C**) Number of SNPs on different continents. SNPs with high frequency (>50%) were comparable among the continents.

SNPs on the Omicron genomes from different continents were analyzed. To exclude the coincidental factors such as sequencing error, we defined mutation site with occurring frequency higher than 1% as an SNP. In total, 130 SNPs were found, among which more than half (72 SNPs) were low-frequency (<10%) and 38 were high-frequency (>50%). The S gene is a hotspot region for mutation, and 41 SNPs were located on it, 14 of which were high-frequency, and 15 SNPs were located in the RBD (Figure 2A). Although structural genes accounted for only one third of the whole genome, their SNPs accounted for more than half of the total, and the S gene accounted for only one eighth of the whole genome, and its SNPs accounted for one fourth (Figure 2B). Spatial distribution showed that South America harbored the greatest number of SNPs (144), followed by North America, Oceania and Europe (137, 128, and 115, respectively). Africa and Europe had a relatively small number of SNPs (92 and 91, respectively). The low-frequency SNPs accounted for a relatively high proportion, which was 40% in Africa and Europe, 44% in Asia, and more than 50% in other continents, while the numbers of high-frequency SNPs exhibited little difference among the continents, which were 39, 44, 42, 38, 38, and 36 in North America, Oceania, Africa, South America, Europe, and Asia, respectively (Figure 2C). 

**Figure 3:**
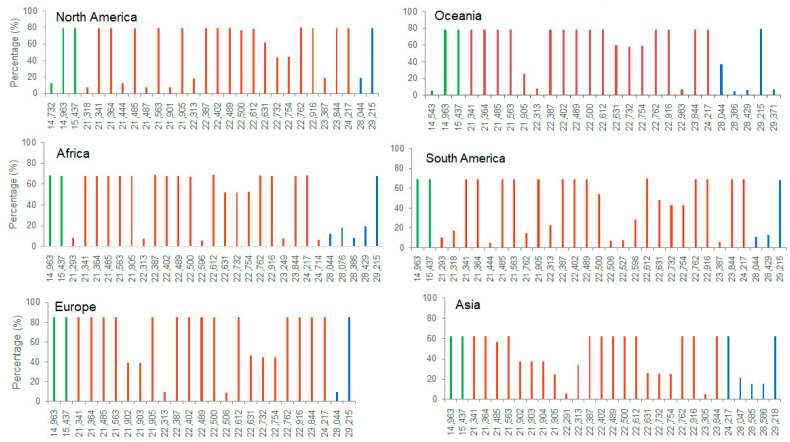
SNPs in specific genes. SNPs with frequency more than 5% in the nsp12, S, and N genes. There were 2, 19, and 2 sites commonly detected in the three genes, respectively. Green bar, SNPs in the nsp12, red bar, SNPs in the S, and blue bar, SNPs in the N.

We also analyzed distributions of SNPs with frequencies more than 5% in the Nsp12, S, and N genes on the six continents (Figure 3). Results showed that Nsp12, encoding the viral RNA-dependent RNA polymerase (RdRp), harbored two SNPs (T14963C and C15437T) with high frequency on all the continents, and two unique SNPs (A14543G and T14732C) were found in Oceania and North America, respectively. Interestingly, all the SNPs in RdRp caused synonymous mutations. In the S gene, the numbers of SNPs in different regions were similar, which was 26 in South America and 21–23 in other continents. Oceania and Africa had 17 high-frequency SNPs, North America had 15, and the other four continents had 14. There were a total 19 SNPs commonly occurring on all six continents. Except nucleotide position 22313, the other 18 commonly occurring SNPs had high-occurring frequencies (>50%), while for the other 18 non-commonly detected ones, the frequencies were relatively low (<20%) except four sites (21,902 and 21,903 in Asia and Europe, 21,904 in Asia, and 22,596 in South America). There were 10 commonly detected and three unique SNPs (22,506, 22,527, and 22,596) located in the RBD. 22,506, 22,527, and 22,596 were detected in South America, 22,506 in Europe, and 22,596 in Africa. All the 10 commonly detected ones exhibited high frequencies, while the three unique ones did not. In the N gene, A28044G and A29215C were commonly detected on the six continents, with A29215C having high-occurring frequency. C28429T was found in Oceania, Africa, and South America. Uniquely detected SNPs (G28386T, C28429T, and C29371T) were found with relatively low frequencies (Figure 3).

### 3.4. Point Mutation Bias Was Found in Omicron Genome

**Figure 4:**
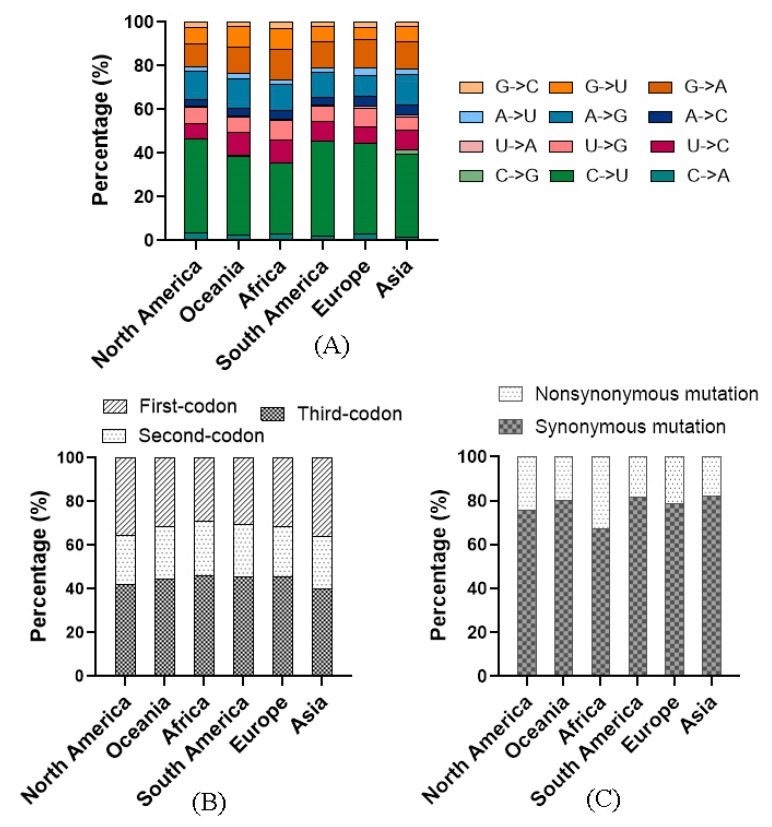
Codon usage of the Omicron variant on the six continents. (**A**) The proportions of the 12 mutational directions. C to U was dominant. (**B**) SNPs on the third codons. The third codon contained the most SNPs. (**C**) Mutation directions on the third codon. Most SNPs on the third codon position caused synonymous mutation.

We analyzed mutation directions of the substituted base and found the pattern was similar among the six continents. Mutation from C to U occurred the most, with frequencies ranging from 33.4% (in Africa) to 43.45% (in South America), accounting for more than one third of the 12 different mutation directions. The frequency of mutations from U to C, A to G, and G to A were all about 10%, while the total frequency of the other eight directions was less than 30% (Figure 4A). Notably, position 22,313 in North America and Africa had two mutation directions: G to A and G to C, leading to amino acid change of R to K and G to C; their occurring frequencies were 85.4% versus 14.6% and 91.7% versus 8.3%, respectively, and position 22,631 in North America and South America also harbored two directions: T to G and T to A, leading to amino acid change of L to R and T and L to Q, with occurring frequency of 69.5% versus 30.5% and 88.9% versus 11.1%, respectively. We also examined the distribution of mutations on the three different codon positions. It was shown that mutation proportions occurring in the third codon position on different continents were all higher than one third: they ranged from 40.2% in Asia to 46.2% in Africa (Figure 4B). It is worth noting that most mutations on the third codon position caused synonymous mutation, especially in Oceania, South America, and Asia, where the proportion of synonymous mutations was more than 80%, followed by North America and Europe, with 75.9% and 78.6%, respectively, while Africa with 67.4% was the lowest (Figure 4C).

### 3.5. Four Groups of SNP Linkages Were Commonly Detected on the Six Continents

We analyzed SNP linkages and their distributions on the six continents. To rule out the possibility that the linkages were caused by occasional factors, the ones containing only two SNPs or frequencies lower than 5% were not calculated in this study. With this criterion, four SNP linkages (I, II, III, and IV) were found on all the six continents. Linkage I contained 38 high-frequency SNPs, the highest number among all, with 1, 20, and 17 located in the intergenic region, nonstructural and structural genes, respectively, among which 14 were on the S gene and 6 in the RBD. All 17 linked SNPs on the structural genes caused nonsynonymous mutations, while 8 of the 20 linked SNPs on the nonstructural genes caused synonymous mutations. Linkage II involved three SNPs, one located in the nsp8 gene causing synonymous mutation and the other two in the S gene causing nonsynonymous mutation. Linkage III involved four SNPs; one caused synonymous mutation and was located in the M gene, and the other three were located in the ORF6 gene and coded the same amino acid, and the mutations caused an amino acid substitution of D to L. Linkage IV involved three SNPs, which were located on the M, ORF6, and ORF7b genes, respectively (Table S1).

**Figure 5:**
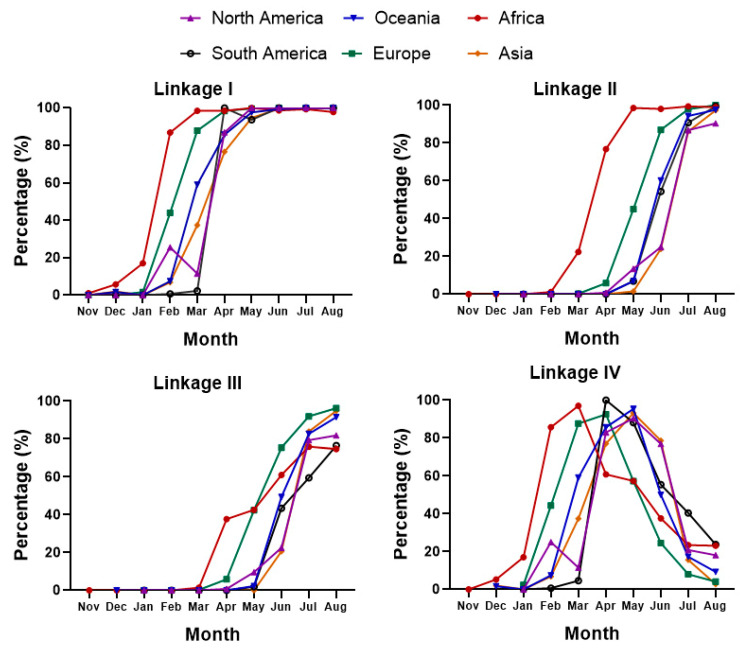
Temporal distributions of the SNP linkages on the six continents. Linkage I contained the most SNPs. The occurring frequencies of linkages I, II, and III remarkably increased over time. Linkage IV only existed four a short time and then decreased sharply.

Temporal distribution revealed that the occurring frequencies of linkages I, II, and III sharply increased in a short time, and then all maintained at a high level, while the occurring frequency of linkage IV increased remarkably, but the high frequency only lasted for two months before it quickly decreased to a very low level. The four linkages were all first detected in Africa, followed by Europe, and the detections in North America, South America, and Asia were the latest. For linkage II, it was detected in Europe two months following being detected in Africa, and the other three were all detected in Europe one month following Africa (Figure 5). In this study, no unique SNP linkage was found on any of the six continents.

### 3.6. Potential Impact of Substitutions in the RBD on Receptor Binding Ability

**Figure 6:**
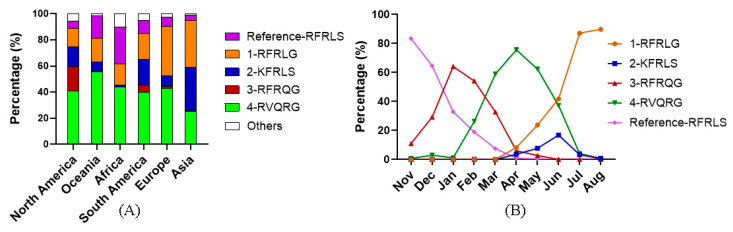
Combinations of the substitutions in the RBD. (**A**) Proportions of different combinations. 1-RFRLG, 2-KFRLS, 3-RFRQG, and 4-RVQRG were the four major combinations. The amino acids corresponding to positions 343, 483, 490, 449, and 493, with position 493 representing four of the six linked amino acids in linkage I. The reference sequence in this study were RFRLS. (**B**) Temporal distributions of the reference virus and the major mutants. The proportion of reference RFRLS decreased over time, while 1-RFRLG increased over time and finally was predominant.

As stated above, ten commonly detected SNPs on the six continents were located in RBD, among which 6 (L368F, T373A, D402N, R405S, S443G, and S493G) were tightly linked and exhibited a high-occurring frequency, while the other four (R343K, L449Q/R, F483V, and R490Q) were not linked together. We calculated the proportions of different amino acid combinations on these substituted sites on the six continents. For convenience, the tightly linked sites were marked together and recorded as 493. The amino acids corresponding to positions 343, 483, 490, 449, and 493 in the reference sequence were RFRLS. We found four major different amino acid combinations, i.e., 1-RFRLG, 2-KFRLS, 3-RFRQG, and 4-RVQRG. Specifically, in South and North America, all four combinations were widely detected, while in Oceania, Europe, and Asia, 1-RFRLG, 2-KFRLS, and 4-RVQRG were the major detections. In Africa, it was 1-RFRLG and 4-RVQRG (Figure 6A). In total, the proportion of reference RFRLS decreased over time, while 1-RFRLG increased over time and finally was definitely predominant, and 3-RFRQG and 4-RVQRG only transiently existed with high-occurring frequency (Figure 6B).

**Figure 7:**
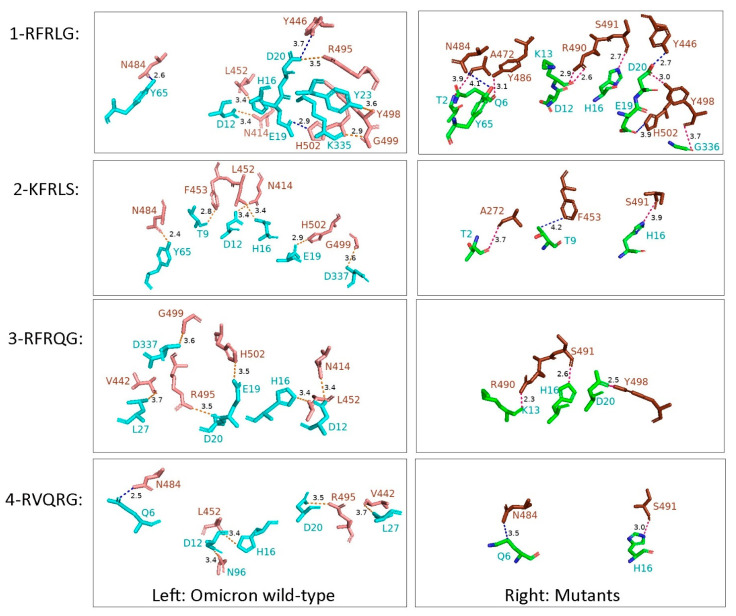
Interaction changes of the four mutants. The mutant 1-RFRLG may slightly have enhanced the interactions between RBD and hACE2, while the other three potentially weakened the interactions. Left column, Omicron wild-type; Right column, mutants. The interactions presented both in wild-type and mutants were not listed, while the changed interactions with the distance <5 Å were marked. Orange bonding stick, interactions only in the wild-type; hot pink bonding stick, interactions only in the mutants; blue bonding stick, changed interactions but within 5 Å both in the wild-type and the mutants.

For the four major combinations of amino acid substitutions in the RBD, we evaluated the impact of their amino acid changes on receptor binding ability. Docking structure revealed that compared with the early Omicron virus (designated as wild-type here), the mutants did not remarkably alter the binding of the virus with the hACE2 receptor. Specifically, for the 1-RFRLG mutant, it only harbored the six-linked amino acid substitutions in linkage I, but compared with wild-type Omicron, there were seven pairs of atoms that weakened their interactions versus eight enhanced, which may slightly have enhanced the receptor binding ability. For the other three mutants (2-KFRLS, 3-RFRQG, and 4-RVQRG), their interaction forces decreased overall, and the pairs of interacting atoms with weakened and enhanced forces were: 6 versus 2, 6 versus 3, and 5 versus 1, respectively (Figure 7). 

## 4. Discussion

The Omicron variant is posing major public health challenges; it is the most heavily mutated among all the pandemic variants [17]. While spreading in populations, the virus continues to inevitably evolve. In this study, we examined large-scale complete-genome sequences of Omicron variants that were collected from different continents to compare evolutionary patterns and further evaluate potential impacts of important mutations on viral biological features.

Previous studies revealed that insertions and deletions are important driving forces contributing to the diversity of SARS-CoV-2, and some are highly relevant to the emergence of variants with changed viral biological or antigenic properties. For example, the 382-nucleotide deletion in Singapore may result in a milder infection [18], the 69–70 deletion in the Alpha variant resulted in an increased spike infectivity [19], and the 156–158 deletion in the Delta variant caused immune escape [9]. In addition, it was demonstrated that different SARS-CoV-2 variants of concern had common and distinctive amino acid substitution patterns, and the Omicron BA.1 exhibited the highest number of recurrent mutations [20]. In this study, we detected nine insertion-and-deletion mutations and found they were concentrated in the Nsp1 gene and NTD of the S1, of which some deletions, such as Del 1, Del 5/8, and Del 7, were only short-lived in a specific area, which may be caused by sequencing errors or other occasional factors. However, Del 4 and 6, resulting in deletions of amino acids 24–26 and 210–212 in the S protein, were widely detected on all the six continents, and their occurring frequencies increased remarkably over time, indicating these two deletions may be caused by host immune pressure and are related to better fitness of the virus. In fact, previous studies demonstrated that deletion of 210–212 at the NTD epitopes resulted in immune escape [8,21]. The biological significance of Del 24–26 needs further investigation. The only insertion with an amino acid occurred in Europe and Asia from May 2022 that, with 100% frequency, could be caused by a bottleneck effect.

SNP calling is important for understanding the genetic variations of the virus. Our previous studies have shown that the Delta variant had a much greater genetic diversity at early stage than at later stage [16,22]. The present study revealed that the Omicron genome harbored fewer SNPs than Delta, it was less diversified, and the number of SNPs varied little among continents, indicating that the virus had a good fitness to humans since its emergence. The number of SNPs with high frequency did not vary much among continents, while the number of SNPs with low frequency differed, which may be caused by environmental or other coincidental factors, rather than related to genetic background differences. The S gene is the mutation hotspot, having the most SNPs per unit length, and the SNPs located in RBD were all nonsynonymous mutations, which may potentially change the viral receptor binding property, but this needs further investigation.

RNA-editing enzyme families in host cells, such as APOBEC and ADAR, are able to induce point mutations to suppress viral infection [23,24]. In this study, we found that the speculated mutation spectrum of the Omicron variant was greatly biased, with the C to U transition dominant. The tendency of mutation biases and context preferences suggested the contribution of the APOBEC, as APOBEC cytosine deaminase enzyme could cause transition of C to U [25]. The C to U bias was also found in other variants of SARS-CoV-2 [16,26]. It was revealed that the proportion of the C to U transition for the Delta variant in different regions was about 36% [16]. However, we found that its proportion for Omicron in Oceania and Asia was also approximately 36%, but in North America, South America, and Europe, the proportions exceeded 41%, and in Africa it was only 32%. The variation among the continents may be caused by different genetic backgrounds of their populations. Moreover, except the SNPs on the intergenic region, the distribution of other SNPs at codon positions was analyzed. Results suggested that proportions of the SNPs in the third codon position on all continents exceeded 40%, implying that the third codons shouldered the most mutation pressures, while the second codons presented the least flexibility as their proportions of the SNPs were all less than 25%. This result was inconsistent with the findings in a previous study that examined the early SARS-CoV-2 strain [27]. Further analysis revealed that SNPs in the third codon position overwhelmingly resulted in synonymous mutations, which may change viral fitness and exposure to natural selection, as synonymous mutations could lead to biological changes through affecting pre-mRNA splicing, translational speed, RNA structure/stability, and so on [28,29,30]. 

It is important to detect co-occurring mutations to study the cooperative manner of different genes at the genetic level [31,32]. Our previous study had shown that a linkage involving 10 SNPs existed on the genome of the Delta variant [16]. In this study, four linkages were detected on the Omicron variant, among which linkage I occurred at a sharply increasing frequency over time and contained a much higher number of SNPs, which was 38, than that of the Delta variant. Combined with the fact that 14 linked SNPs in linkage I were located in the S and 6 were in the RBD, we inferred that though Omicron had great changes in antigenicity and a remarkable ability to evade immunity [1,33], it was still under a great immune pressure from the host. No unique linkages were detected among the continents, revealing factors such as genetic backgrounds difference, climate variation, and so on, having little effect on the evolution of the Omicron variant.

The amino acid changes in SARS-CoV-2 RBD would potentially affect viral biological properties [34,35]. The interactions between the major amino acid substitutions in the S-RBD and hACE2 have been predicted by computational methods in this study. It was reported that 17 amino acids at positions 417, 446, 449, 453, 455, 456, 475, F486, 487, 489, 493, 496, 498, 500, 501, 502, and 505 are responsible for hACE2 identification and interaction by H bonds and salt bridges [36], and Omicron shared a highly similar over binding to the hACE2 receptor [37]. Among the ten substitutions in the RBD detected in this study, L449Q/R and S493G could directly change the host–virus interaction as they make direct contact with hACE2, while the other eight substitutions that do not directly have contact with the receptor may also affect the interactions by altering the spatial conformation of the adjacent amino acids. Our results suggested that the six amino acid substitutions at linkage I slightly enhanced the binding ability of the RBD to hACE2, while in combination with other substitution in the RBD, such as F483V, R490Q, and L493Q/R, the interactions were potentially attenuated. The proportion of the mutant (1-RFRLG) with slightly enhanced binding ability increased over time and finally was predominant, indicating that this mutant exhibited a better fitness. Moreover, the mutants with potentially weakened interaction distributed widely on the six continents, suggesting that the amino acid change may be caused by immune pressure. However, the changed interactions in this study did not mean that the virus changed its ability to infect host cells. Usually, stronger binding would not imply higher functionality, as for SARS-CoV-2; besides attachment to the receptor, other factors that affect membrane fusion, such as cleavage efficiency with furin and TMPRSS2, are also critical for the virus entry. A recent study has revealed that the Omicron variant had altered the entry pathway preference, probably causing alterations in tissue tropism [38]. 

In conclusion, this study revealed genetic variation signatures on the genome of the Omicron variant during viral transmission. The results suggested that the Omicron genome was relatively stable, it contained small number of SNPs, and all the high-frequency SNPs were tightly linked. A significant proportion of the SNPs underwent strong immune pressure and occurred on the S-RBD, which may potentially change the biological properties of the virus. There is still much to learn about the function and mechanism of RBD substitutions and SNP linkages. Due to the sampling differences and limitation of sequencing capacity, the conclusions in this study may be biased. Nevertheless, this study tracked mutation dynamics of the Omicron temporally and spatially, and it could provide scientific data for the control and prevention of SARS-CoV-2.

## Figures and Tables

**Table 1:** Deletions/insertions on the SARS-CoV-2 Omicron genome.

	Genomic Position	Amino Acid	Protein	Protein Position	Continent	Frequency (%)
Del 1	247–255	HVM	NSP1	83–85	Africa	3.77
Del 2	244–258	GHVMV	NSP1	82–86	Oceania	2.08
					Africa	3.59
					Europe	1.70
					Asia	1.32
Del 3	421–429	KSF	NSP1	141–143	North America	4.48
					Africa	19.17
					South America	13.06
					Europe	4.40
					Asia	2.18
Del 4	21,355–21,363	LPP	S	24–26	North America	79.06
					Oceania	78.99
					Africa	67.38
					South America	68.90
					Europe	85.00
					Asia	62.32
Del 5	21,472–21,486	TWFHA	S	63–67	Asia	12.89
Del 6	21,910–21,918	EPE	S	210–212	North America	80.20
					Oceania	79.69
					Africa	71.15
					South America	78.98
					Europe	86.89
					Asia	65.59
Del 7	21,742–21,837	ϖ	S	153–184	South America	13.79
Del 8	21,907–21,909	V	S	207	Asia	12.89
Inser 1	21,904–21,906	L	S	207 *	Europe	38.69
					Asia	37.35

Note: Del = Deletion. Inser = Insertion. ϖ = RVYSSANNCTFEYVSQPFLMDLEGKQGNFKNL. * = Between amino acid 206 and 207 of the S protein. The two deletions (Del and Del 8) marked in color were tightly linked.

## Data Availability

No new data were created or analyzed in this study. Data sharing is not applicable to this article.

## Supplementary Materials

The following supporting information can be downloaded at: https://www.mdpi.com/article/10.3390/v15020321/s1, Figure S1: Number of sequences in different continents. Table S1: Specific sites of the four groups of SNP linkages that commonly detected on the six continents.

## References

[1] Hu J., Peng P., Cao X., Wu K., Chen J., Wang K., Tang N., Huang A.L. (2022). Increased immune escape of the new SARS-CoV-2 variant of concern Omicron. Cell. Mol. Immunol..

[2] Karim S.S.A., Karim Q.A. (2021). Omicron SARS-CoV-2 variant: A new chapter in the COVID-19 pandemic. Lancet.

[3] Wang Q., Guo Y., Iketani S., Nair M.S., Li Z., Mohri H., Wang M., Yu J., Bowen A.D., Chang J. (2022). Antibody evasion by SARS-CoV-2 Omicron subvariants BA.2.12.1, BA.4 and BA.5. Nature.

[4] Iketani S., Liu L., Guo Y., Liu L., Chan J.F.W., Huang Y., Wang M.L., Yang Y., Jian C., Hu H. (2022). Antibody evasion properties of SARS-CoV-2 Omicron sublineages. Nature.

[5] Liu L., Iketani S., Guo Y., Chan J.F.W., Wang M., Liu L., Luo Y., Chu H., Huang Y., Nair M.S. (2022). Striking antibody evasion manifested by the Omicron variant of SARS-CoV-2. Nature.

[6] Schubert M., Bertoglio F., Steinke S., Heine P.A., Ynga-Durand M.A., Maass H., Sammartino J.C., Cassaniti I., Zuo F., Du L. (2022). Human serum from SARS-CoV-2-vaccinated and COVID-19 patients shows reduced binding to the RBD of SARS-CoV-2 Omicron variant. BMC Med..

[7] Xie X., Liu Y., Liu J., Zhang X., Zou J., Fontes-Garfias C.R., Xia H., Swanson K.A., Cutler M., Cooper D. (2021). Neutralization of SARS-CoV-2 spike 69/70 deletion, E484K and N501Y variants by BNT162b2 vaccine-elicited sera. Nat. Med..

[8] McCarthy K.R., Rennick L.J., Nambulli S., Robinson-McCarthy L.R., Bain W.G., Haidar G., Duprex W.P. (2021). Recurrent deletions in the SARS-CoV-2 spike glycoprotein drive antibody escape. Science.

[9] McCallum M., De Marco A., Lempp F.A., Tortorici M.A., Pinto D., Walls A.C., Veesler D. (2021). N-terminal domain antigenic mapping reveals a site of vulnerability for SARS-CoV-2. Cell.

[10] Liu L., Wang P., Nair M.S., Yu J., Rapp M., Wang Q., Luo Y., Chan J.F.W., Sahi V., Figueroa A. (2020). Potent neutralizing antibodies against multiple epitopes on SARS-CoV-2 spike. Nature.

[11] Yao H., Lu X., Chen Q., Xu K., Chen Y., Cheng M., Chen K., Cheng L., Weng T., Shi D. (2020). Patient-derived SARS-CoV-2 mutations impact viral replication dynamics and infectivity in vitro and with clinical implications in vivo. Cell Discov..

[12] Oude Munnink B.B., Worp N., Nieuwenhuijse D.F., Sikkema R.S., Haagmans B., Fouchier R.A., Koopmans M. (2021). The next phase of SARS-CoV-2 surveillance: Real-time molecular epidemiology. Nat. Med..

[13] Morais I.J., Polveiro R.C., Souza G.M., Bortolin D.I., Sassaki F.T., Lima A.T.M. (2020). The global population of SARS-CoV-2 is composed of six major subtypes. Sci. Rep..

[14] Justo Arevalo S., Zapata Sifuentes D., Huallpa C.J., Landa Bianchi G., Castillo Chávez A., Garavito-Salini Casas R., Uceda-Campos G., Pineda Chavarria R. (2021). Global Geographic and Temporal Analysis of SARS-CoV-2 Haplotypes Normalized by COVID-19 Cases During the Pandemic. Front. Microbiol..

[15] Elbe S., Buckland-Merrett G. (2017). Data, disease and diplomacy: GISAID’s innovative contribution to global health. Glob Chall..

[16] Zhang J., Fan L., Xu H., Fu Y., Peng X., Zheng Y., Yu J., He J. (2022). Evolutionary Pattern Comparisons of the SARS-CoV-2 Delta Variant in Countries/Regions with High and Low Vaccine Coverage. Viruses.

[17] Otto S.P., Day T., Arino J., Colijn C., Dushoff J., Li M., Ogden N.H. (2021). The origins and potential future of SARS-CoV-2 variants of concern in the evolving COVID-19 pandemic. Curr. Biol..

[18] Young B.E., Fong S.W., Chan Y.H., Mak T.M., Ang L.W., Anderson D.E., Lee C.Y.-P., Amrun S.N., Lee B., Goh Y.S. (2020). Effects of a major deletion in the SARS-CoV-2 genome on the severity of infection and the inflammatory response: An observational cohort study. Lancet.

[19] Meng B., Kemp S.A., Papa G., Datir R., Ferreira I., Marelli S., Harvey W.T., Lytras S., Mohamed A., Galled G. (2021). Recurrent emergence of SARS-CoV-2 spike deletion H69/V70 and its role in the Alpha variant B.1.1.7. Cell Rep..

[20] Nikolaidis M., Papakyriakou A., Chlichlia K., Markoulatos P., Oliver S.G., Amoutzias G.D. (2022). Comparative Analysis of SARS-CoV-2 Variants of Concern, Including Omicron, Highlights Their Common and Distinctive Amino Acid Substitution Patterns, Especially at the Spike ORF. Viruses.

[21] Liu X., Guo L., Xu T., Lu X., Ma M., Sheng W., Peng H., Cao L., Zheng F., Huang S. (2021). A comprehensive evolutionary and epidemiological characterization of insertion and deletion mutations in SARS-CoV-2 genomes. Virus Evol..

[22] Fan L.Q., Hu X.Y., Chen Y.Y., Peng X.L., Fu Y.H., Zheng Y.P., Yu J.-M., He J.-S. (2021). Biological Significance of the Genomic Variation and Structural Dynamics of SARS-CoV-2 B.1.617. Front. Microbiol..

[23] Deng S., Xing K., He X. (2022). Mutation signatures inform the natural host of SARS-CoV-2. Natl. Sci. Rev..

[24] Di Giorgio S., Martignano F., Torcia M.G., Mattiuz G., Conticello S.G. (2020). Evidence for host-dependent RNA editing in the transcriptome of SARS-CoV-2. Sci. Adv..

[25] Simmonds P. (2020). Rampant C-->U Hypermutation in the Genomes of SARS-CoV-2 and Other Coronaviruses: Causes and Consequences for Their Short- and Long-Term Evolutionary Trajectories. mSphere.

[26] Tonkin-Hill G., Martincorena I., Amato R., Lawson A.R.J., Gerstung M., Johnston I., Jackson D.L., Park N., Lensing S.V., Quail M.A. (2021). Patterns of within-host genetic diversity in SARS-CoV-2. Elife.

[27] Teng X., Li Q., Li Z., Zhang Y., Niu G., Xiao J., Yu J., Zhang Z., Song S. (2020). Compositional Variability and Mutation Spectra of Monophyletic SARS-CoV-2 Clades. Genom. Proteom. Bioinform..

[28] Ingvarsson P.K. (2010). Natural selection on synonymous and nonsynonymous mutations shapes patterns of polymorphism in Populus tremula. Mol. Biol. Evol..

[29] Hanson G., Coller J. (2018). Codon optimality, bias and usage in translation and mRNA decay. Nat. Rev. Mol. Cell Biol..

[30] Supek F., Minana B., Valcarcel J., Gabaldon T., Lehner B. (2014). Synonymous mutations frequently act as driver mutations in human cancers. Cell.

[31] Qin L., Ding X., Li Y., Chen Q., Meng J., Jiang T. (2021). Co-mutation modules capture the evolution and transmission patterns of SARS-CoV-2. Brief. Bioinform..

[32] Chen H., Zhou X., Zheng J., Kwoh C.K. (2016). Rules of co-occurring mutations characterize the antigenic evolution of human influenza A/H3N2, A/H1N1 and B viruses. BMC Med. Genomics.

[33] Ao D., Lan T., He X., Liu J., Chen L., Baptista-Hon D.T., Zgang K., Wei X. (2022). SARS-CoV-2 Omicron variant: Immune escape and vaccine development. MedComm.

[34] Zhang Z., Zhang J., Wang J. (2022). Surface charge changes in spike RBD mutations of SARS-CoV-2 and its variant strains alter the virus evasiveness via HSPGs: A review and mechanistic hypothesis. Front. Public Health.

[35] Harvey W.T., Carabelli A., Jackson B., Gupta R., Thomson E.C., Harrison E.M., Ludden C., Reeve R., Rambaut A., Peacock S. (2021). SARS-CoV-2 variants, spike mutations and immune escape. Nat. Rev. Microbiol..

[36] Lan J., Ge J., Yu J., Shan S., Zhou H., Fan S., Zhang Q., Shi X., Wang Q., Zhang L. (2020). Structure of the SARS-CoV-2 spike receptor-binding domain bound to the ACE2 receptor. Nature.

[37] Lan J., He X., Ren Y., Wang Z., Zhou H., Fan S., Zhu C., Liu D., Shao B., Liu T.-Y. (2022). Structural insights into the SARS-CoV-2 Omicron RBD-ACE2 interaction. Cell Res..

[38] Willett B.J., Grove J., MacLean O.A., Wilkie C., De Lorenzo G., Furnon W., Cantoni D., Scott S., Logan N., Ashraf S. (2022). SARS-CoV-2 Omicron is an immune escape variant with an altered cell entry pathway. Nat. Microbiol..

