# Supplementary figures and images for: Tracking of Mutational Signature of SARS-CoV-2 Omicron on Distinct Continents and Little Difference was Found
Source: Viruses. 2023 Jan 23;15(2):321. doi: 10.3390/v15020321 (PMC9967123; doi:10.3390/v15020321)

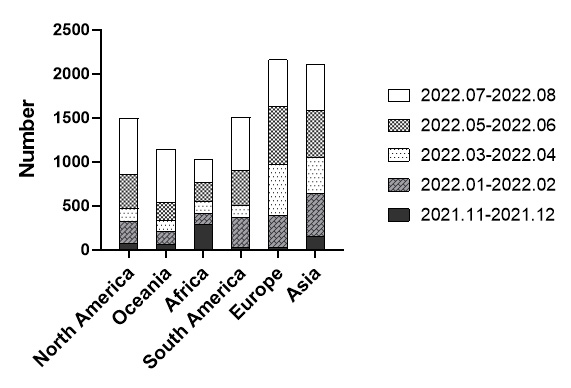

Supplement: Supplementary file 1 [file viruses-15-00321-s001.zip › Figure S1.jpg]
